# Applying different levels of practice variability for motor learning: More is not better

**DOI:** 10.7717/peerj.17575

**Published:** 2024-06-25

**Authors:** Carla Caballero, David Barbado, Manuel Peláez, Francisco J. Moreno

**Affiliations:** 1 Sport Sciences Department, Sport Research Centre, Universiad Miguel Hernández de Elche, Elche, Alicante, Spain; 2 Neurosciences Research Group, Alicante Institute for Health and Biomedical Research (ISABIAL), Spain, Alicante, Spain

**Keywords:** Variable practice load, Skill acquisition, Learning process

## Abstract

**Background:**

Variable practice is a broadly used tool to improve motor learning processes. However, controversial results can be found in literature about the success of this type of practice compared to constant practice. This study explored one potential reason for this controversy: the manipulation of variable practice load applied during practice and its effects according to the initial performance level and the initial intrinsic variability of the learner.

**Method:**

Sixty-five participants were grouped into four practice schedules to learn a serial throwing task, in which the training load of variable practice was manipulated: one constant practice group and three groups with different variable practice loads applied. After a pre-test, participants trained for 2 weeks. A post-test and three retests (96 h, 2 weeks and 1 month) were carried out after training. The participants’ throwing accuracy was assessed through error parameters and their initial intrinsic motor variability was assessed by the autocorrelation coefficient of the error.

**Results:**

The four groups improved their throwing performance. Pairwise comparisons and effect sizes showed larger error reduction in the low variability group. Different loads of variable practice seem to induce different performance improvements in a throwing task. The modulation of the variable practice load seems to be a step forward to clarify the controversy about its benefits, but it has to be guided by the individuals’ features, mainly by the initial intrinsic variability of the learner.

## Introduction

Variable practice has been suggested for years as a useful tool to enhance learning processes. The Schema Theory suggests that this type of practice provides an increased schema that allows the appropriate transfer and generalization to the different demands of a continuously changing environment (Schmidt, 1975). Nevertheless, not all the studies have found positive results after variable practice. Some works have shown that constant practice displays better (Breslin et al., 2012; Edwards & Hodges, 2012; Zipp & Gentile, 2010) or similar results (Czyż, 2021; Reynoso et al., 2013; van den Tillaar & Marques, 2013; Willey & Liu, 2018) than variable practice mainly in closed motor skills, when motor behavior can be planned and the stabilization of the motor pattern is required (Van Rossum, 1990). This affirmation has been evidenced in the so-called especial skills (Breslin et al., 2012; Keetch et al., 2005), which are skills with an advantage in performance result from massive amounts of practice (Czyz et al., 2013; Keetch et al., 2005; Simons et al., 2009). In these especial skills the specificity process seems to be stronger than generalization (Czyz & Moss, 2016). Thus, constant practice would be more appropriate when the conditions under which the information or skills were practiced are identical to those in the test (Keetch et al., 2005). Nevertheless, no environment is entirely predictable, and if it were, no equal actions would be possible because intrinsic motor variability cannot be avoided (Dhawale, Smith & Ölveczky, 2017; Faisal, Selen & Wolpert, 2008; Renart & Machens, 2014; Stein, Gossen & Jones, 2005). Then, variable practice could still be useful in improving motor performance in highly predictable conditions compared to constant practice. Several studies have addressed this question, and, aside from the works related to especial skills, some of them support that practicing under variable conditions improves task performance in close task to a higher extent than constant practice, mainly in terms of retentions and transfer (Asencio-Alonso, Gea García & Menayo Antúnez, 2021; Hernández-Davo et al., 2014; Lee, Magill & Weeks, 1985; Shapiro & Schmidt, 1982; Shea & Kohl, 1990; Van Rossum, 1990; Williams & Hodges, 2005; Zetou et al., 2014).

The central nervous system (CNS) regulates intrinsic motor variability in order to ease the exploration of the large number of possible configurations offered by the many motor system degrees of freedom (DOF) that can lead to a desired solution, playing an important role for motor learning and the ability to adapt (Barbado et al., 2012; Davids et al., 2003; Hu et al., 2020; Lamoth & van Heuvelen, 2012; Mandelblat-Cerf, Paz & Vaadia, 2009; Moreno & Ordoño, 2010; Wu et al., 2014). Then, the explanation given for variable practice being useful in both predictable and unpredictable contexts is that, when the individual is not displaying enough intrinsic variability movement to achieve an adequate control or adaptation, variability practice could be used to promote those exploratory behaviors (Ranganathan & Newell, 2013). This is, variable practice would challenge the learner with a variety of movements that cover a wide range of possible motor solutions for a specific task, which could be modulated by the practitioner, even when the task conditions, apparently, remain unchanged (Moreno & Ordoño, 2015). Another hypothesis is that increasing learners’ motor variability by using variable practice would increase the learners’ sensitivity to motor error, which helps them cope with the inherent motor fluctuations of any movement (Barbado et al., 2017).

Considering the above-mentioned rationale, some authors suggest that the individual characteristics and task constraints are key factor in modulating the effectiveness of variable practice during learning (Caballero et al., 2017; Newell & Vaillancourt, 2001; Vaillancourt & Newell, 2002, 2003). Some works have already specified the need to adapt the level of variable practice to the learner’s level, suggesting that variable and constant practice have a differential effect on learning, depending on individual characteristics (García-Herrero et al., 2016; Taheri, Fazeli & Poureghbali, 2017). Some studies have proved that learners displayed different levels of intrinsic variability according to their learning stage, showing high intrinsic variability when motor exploration is required to learn a novel task and low intrinsic variability when trying to improve their performance by exploiting a stable and viable motor solution (Woolley & Doupe, 2008; Wu et al., 2014). Consequently, we argue that the controversial results were probably caused by the fact that the practice load (understood as the amount of variability applied into the task conditions) was not adjusted according to the learner’s individualities, such as the amount of intrinsic motor variability.

According to McEwen & Lasley (2002), the body’s response to a training load would follow an inverted “U” shape, in which very low load levels will not be enough to cause adaptations, just as very high levels could be detrimental, causing non-desired adaptations. This idea was also taken by Moreno & Ordoño (2015), who extrapolated it to the concept of variable practice load in motor learning. Thus, some authors have proposed a moderate load of variable practice as the optimal variable load to obtain positive results (Caballero, Luis & Sabido, 2012; Harbourne & Stergiou, 2009; Ranganathan & Newell, 2010). From the authors’ point of view, this idea may be directly related to the Challenge-point framework proposed by Guadagnoll & Lee (2004), which suggest that there is an optimal challenge point that allows learning to acquire information from the environment thanks to practice conditions. However, most of the studies in the current literature have compared constant practice *versus* variable practice. Some studies under the contextual interference paradigm have analyzed the effect of different variability scheduling, suggesting that gradual increases in contextual interference seem more effective for enhancing motor learning (Porter & Beckerman, 2016; Porter & Magill, 2010). However, as far as the authors’ know, there are no experiments that have compared different levels of variability in other dimensions such as heterogeneity or numerosity of variability (Raviv, Lupyan & Green, 2022).

The main aim of the present study was to assess which amount of variability is better for novice participants to learn a discrete throwing task. It was hypothesized that the amount of variable practice (practice load) and the amount of learning would show an inverted “U” shape relationship. Specifically, constant practice (or the lowest level of variable practice) will induce low levels of motor learning as it will not provide a sufficient practice stimulus to maximize motor learning. High levels of variable practice will be an excessive practice load that will not maximize learning either. Thus, moderate levels of variable practice will be the practice load that will show the highest amount of learning. As a *post-hoc* analysis, the experiment will focus on how the potential effect of the different practice conditions can also be modulated by the participants’ initial performance level and their initial intrinsic variability. To test the above-mentioned hypotheses, the load of variable practice was manipulated in this work through the heterogeneity of the throwing movements (Raviv, Lupyan & Green, 2022). Heterogeneity was chosen for this study because it has been proposed as the main predictor of learning and generalization (Bowman & Zeithamova, 2020; Poletiek & van Schijndel, 2009; Schiff et al., 2021), being sometimes considered the most relevant source of variability (Raviv, Lupyan & Green, 2022). The participants were encouraged to throw in different orientations and angles by dispersing the location of the targets. Therefore, the variable practice groups practiced with increased heterogeneity of the throws according to target locations, going from less (low variability group) to more spread apart (high variability group). It must be pointed out that the numerosity source of variability was not manipulated since, apart from the constant group, which practiced with only one target location, the variability groups kept the number of targets constant.

## Materials and Methods

### Participants

Sixty-six healthy participants (33 females, 33 males) took part in this study (age = 23.35 ± 3.9 years; height = 1.7 ± 0.1 m; mass = 66.3 ± 11.9 Kg). Exclusion criteria included current musculoskeletal injuries or coordination deficits that impaired participants from throwing a ball against a wall in front of them and catching the rebound. All participants were right-handed and participated in the study voluntarily.

Written informed consent was obtained from each participant prior to testing. Data were treated anonymously, and all participants were informed of the risks and benefits of the trial. The experimental procedures used in this study were in accordance with the Declaration of Helsinki and were approved by the University of Miguel Hernández of Elche Office for Research Ethics (Ethical Application Ref: DPS.FMH.01.16, DCD.FMH.01.21).

### Experimental procedure and data collection

**Figure 1:**
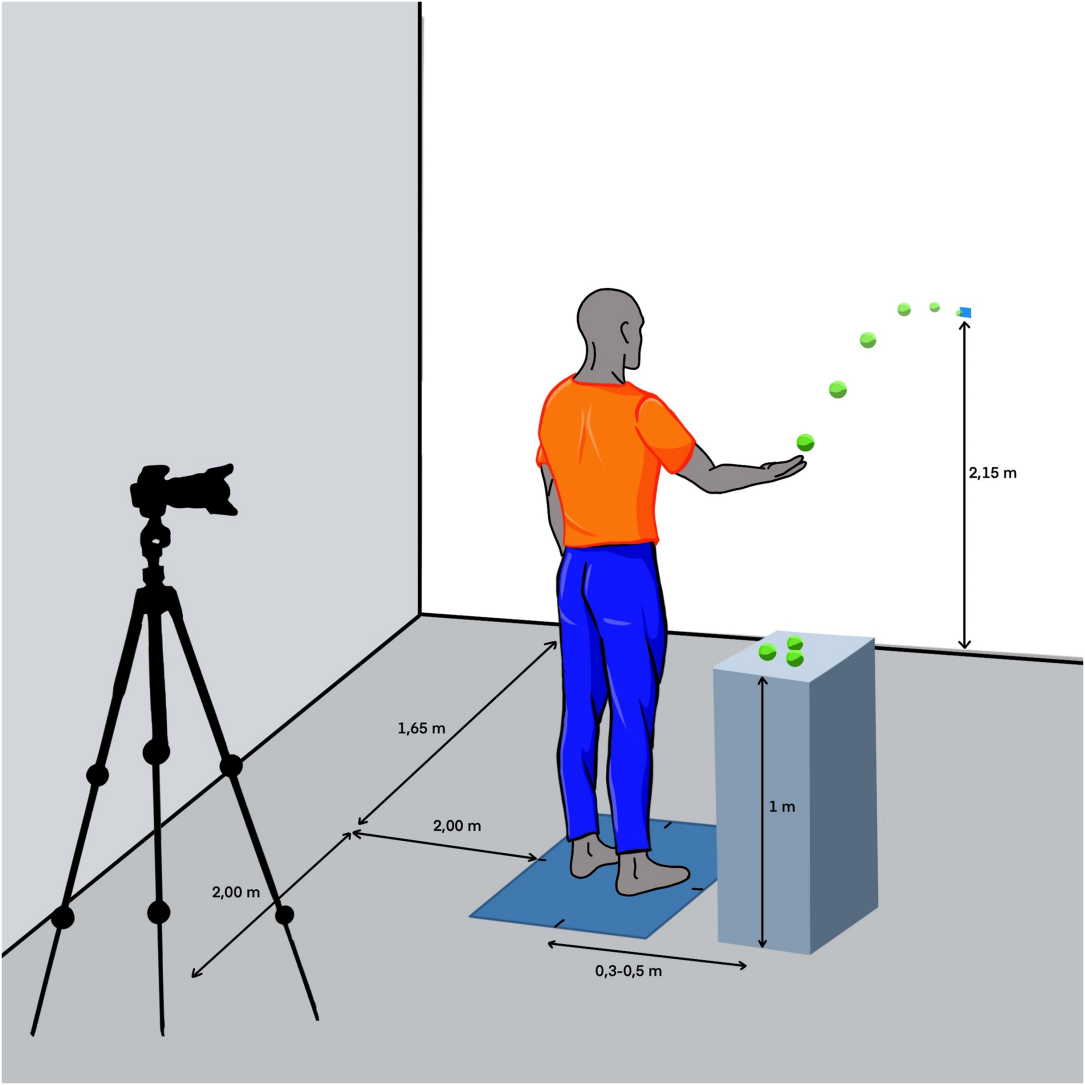
Schematic illustration of the protocol setup. Created using Procreate iPad OS 5.2.9.

Participants performed a repetitive underhand throwing test with a tennis ball using their right hand. The task goal was to hit a target located on a wall that was at 1.65 m from the participant and at a height of 2.15 m high from the ground. During the throws, the participants stood with their feet placed at shoulder width and the foot orientation was such that the vector formed by the heels was parallel to the mediolateral axis of the platform (see Fig. 1). Participants rested their left hand on their hip during the entire test. After a throw, the participants had to catch the tennis ball as it bounced off the wall to perform the upcoming throw, using their right hand all the time. In case participants did not catch the ball, they could catch a new ball located on their right side at hip height (see Fig. 1). All the participants kept a rhythm of 40 throws per minute during the throws following a beep given by a metronome.

The protocol carried out by all participants consisted of a pre-test, a training period of 2 weeks, a post-test, and three retests (96 h, 2 weeks and 1 month after training period). Each test (pre-test, post-test, and retest) contained four sets of 20 repetitions each. The sets were divided into two blocks of two sets, with a 30 s rest between sets and 1 min rest between blocks. The training period consisted of eight sessions of eight sets of 20 trials each. The sets were divided into blocks of four sets. The resting time between sets was 30 s and the resting time between blocks was 1 min.

**Figure 2:**
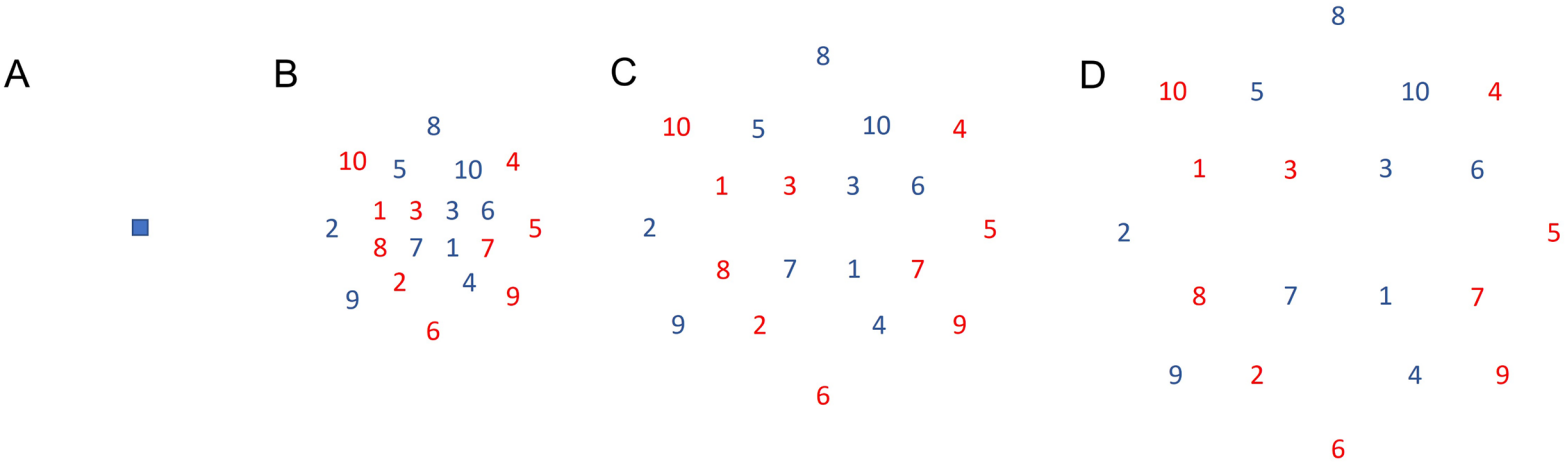
Targets. (A) Representation of the target used for the tests and for the constant group during practice; (B) Representation of the targets used for the low variability group during practice. The maximum radius was 20 cm (6.91°); (C) Representation of the targets used for the medium variability group during practice. The maximum radius was 40 cm (13.63°); (D) Representation of the targets used for the high variability group during practice. The maximum radius was 60 cm (19.98°).

After the pre-test, participants were distributed according to their initial performance to homogenize the group’s performance level into four experimental groups and a control group, which did not practice in any condition (*N = 10*). The four experimental groups were designed according to the manipulation of the load of variable practice, referred to the amount of variable practice induced in the task conditions according to the heterogeneity of the throwing movements (Raviv, Lupyan & Green, 2022), as follows: a) constant group (CS), which trained doing constant practice in the same conditions as the evaluation tests explained above (*N = 14*); b) low variability group (LV), which trained the throws with variable angles and orientation, aimed to targets dispersed in a radius from 0 to of 20 cm (6.91°) maximum from the main target (*i.e*., the target used during the evaluation tests) (*N = 13*); c) medium variability group (MV), which throwed with more variable angles and orientations trying to hit targets dispersed in a radius from 0 to 40 cm (13.63°) from the main target (*N = 11*); and, d) high variability group (HV), which throwed with variable angles and orientations aimed to targets dispersed in a range of variable radius up to 60 cm (19.98°) maximum from the main target (*N = 15*). For all the variable groups, twenty targets were scattered across the circle within the corresponding maximum radius. The targets were spread out randomly and numbered to guide the participants during the practice series. In order to reduce the attentional demands of the task during variable practice, and due to the large variety of possible trajectories practiced in variable groups, the targets were distributed in two sets numbered from 1 to 10 and in two colors, red and blue, going in numerical sequence. The participants were instructed to start with targets colored in red and then those colored in blue. No participant reported any difficulty in identifying the targets or the order (see Fig. 2 to check the target features).

Each ball impact on the wall was video recorded with a “Sony HDR-SR8E” digital camera (Sony, Tokyo, Japan), at a frequency of 50 Hz, to establish the impact zone of each throw.

### Data analysis and reduction

**Figure 3:**
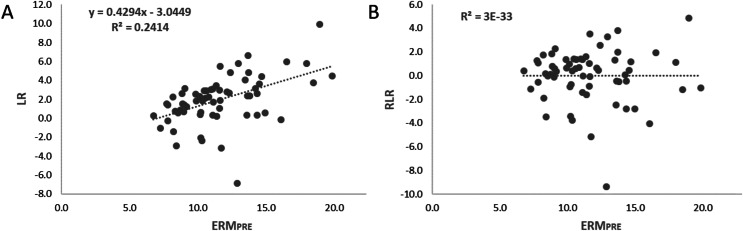
Representation of the steps followed in computing the residual amount of learning (RAL). (A) Linear relationship between the initial performance (MRE_PRE_) and the absolute amount of learning (AAL); (B) Linear relationship between the initial performance (MRE_PRE_) and the residual amount of learning (RAL) computed by subtracting the predicted amount of learning to the real amount of learning.

The ball impacts were digitalized using Kinovea (version 0.8.15). An *ad-hoc* Matlab (version 7.11; Mathworks, Natick, MA, USA) routine was used for the calculation of real-space Cartesian coordinates of the ball rebounds. The video analysis to identify the impact zone was made manually by two different trained researchers, following the procedure used in previous studies (Moreno et al., 2023, 2020). Each participant’s accuracy was measured through the mean radial error (MRE), as a global accuracy index, computed as the average of the vector distance magnitude (cm) of the ball impact place from the target position of all the throws. The average of the absolute error in the vertical (AE_V_) and horizontal (AE_H_) axis were also calculated and included in the Supplemental Material. In order to compute the absolute amount of learning (AAL) the differences between the post-test and each of the three retention tests compared to the pre-test were calculated. The computations were made as follows: a) differences between pre-test (MRE_PRE_) and post-test (MRE_POST_); b) differences between pre-test (MRE_PRE_) and retest 1 (MRE_RET1_); c) differences between pre-test (MRE_PRE_) and retest 2 (MRE_RET2_); d) differences between pre-test (MRE_PRE_) and retest 3 (MRE_RET3_). However, Pearson’s correlations revealed that the different ways to compute the absolute amount of learning by the differences between the post-test and the three retention tests compared to the pre-test were positively correlated with each other (Table 1). Thus, to simplify the results, it was decided to use the amount of learning computed by the differences in MRE between the pre-test and the retest 3, representing learning after 1 month. In addition, the correlational analysis revealed a relationship between the initial performance and the absolute amount of learning (r = 0.308*, *p* < 0.05). This is, participants who displayed lower performance (higher MRE) achieved higher absolute amount of learning (higher AAL). Then, to avoid the potential bias on the amount of learning caused by the initial performance, a linear regression method was computed following the procedure used in previous studies (Barbado et al., 2017; Caballero, Moreno & Barbado, 2021). For that, a linear regression was estimated between initial performance (MRE_PRE_) and the absolute amount of learning (AAL) (see Fig. 3A). Finally, participants’ residual scores were used to compute the residual amount of learning (RAL), which was used as the amount of learning index (Fig. 3B).

**Table 1:** Correlations between the different absolute amount of learning indexes computed.

	MRE_PRE_ – MRE_POST_	MRE_PRE_ – MRE_RET1_	MRE_PRE_ – MRE_RET2_
MRE_PRE_ – MRE_RET1_	0.882*		
MRE_PRE_ – MRE_RET2_	0.598*	0.559*	
MRE_PRE_ – MRE_RET3_	0.756*	0.774*	0.544*

**Note:**

* *p* < 0.001.

In this study, the standard deviation of the ball impact position was not used as the participant’s initial intrinsic variability index because it was highly correlated (r > 0.9) with error parameters. Instead, the Lag-1 autocorrelation coefficient of the time series data from the vertical and horizontal positions of the ball impacts in the pre-test was calculated to assess the initial intrinsic variability of the participants. Lag-1 autocorrelation coefficient allows for addressing the relationship between consecutive attempts in tasks, such as hitting a target, and assessing how each attempt is influenced by the one before it. The autocorrelation function measures the average strength of each data point with data point k time steps away (Hunt, 2016). This approach is supported by the study of Skurvydas, Brazaitis & Kamandulis (2010), who applied autocorrelation as a measure of the linear dependencies of successive trials, reflecting the variability of motor outcomes. Therefore lag-1 autocorrelation is valued for providing insights into the corrections a participant makes over successive trials, offering a complement to conventional accuracy and variability measurements. The average of the autocorrelation coefficient of the positions of the ball impacts on each axis was determined as a global variable of each participant’s initial intrinsic variability.

### Statistical analysis

Prior to any statistical analysis, three subjects were removed from the original sample due to measurement issues. Thus, the participants analyzed were 63, distributed in the different groups as it was specified above in the Experimental procedure and Data Collection. The normality of the variables was evaluated using the Kolmogorov-Smirnov test with the Lilliefors correction. Intention-to-treat analyses were carried out for all parameters. Only one participant from the control group did not complete all the measurements. Specifically, that participant could not perform retest 1, so missed data were imputed following a multiple imputation method based on a linear regression estimation. A mixed ANOVA was conducted for accuracy, with *practice* as a within-subject factor (five levels: pre-test, post-test, retest 1, retest 2, retest 3) and *group* as a between-subject factor (five levels: control, constant, low variability, medium variability, high variability). A Bonferroni adjustment for multiple comparisons was performed to ascertain differences between groups for the different test evaluations. Hedge’s *g* was calculated as the effect size index. Hedge’s *g* algorithm adjusts Cohen’s *d* score, which is based on sample averages and gives a biased estimate of the population effect size (Hedges & Olkin, 1985), especially for small samples (*N* < 20). Values of effect sizes ≥0.8 were considered large, from 0.8 to 0.5 were considered moderate, and <0.5 were considered small effect sizes (Cohen, 1988). Pearson’s correlations were carried out to extract underlying relationships between the initial intrinsic variability, the level of variable practice load, and the amount of learning.

## Results

**Figure 4:**
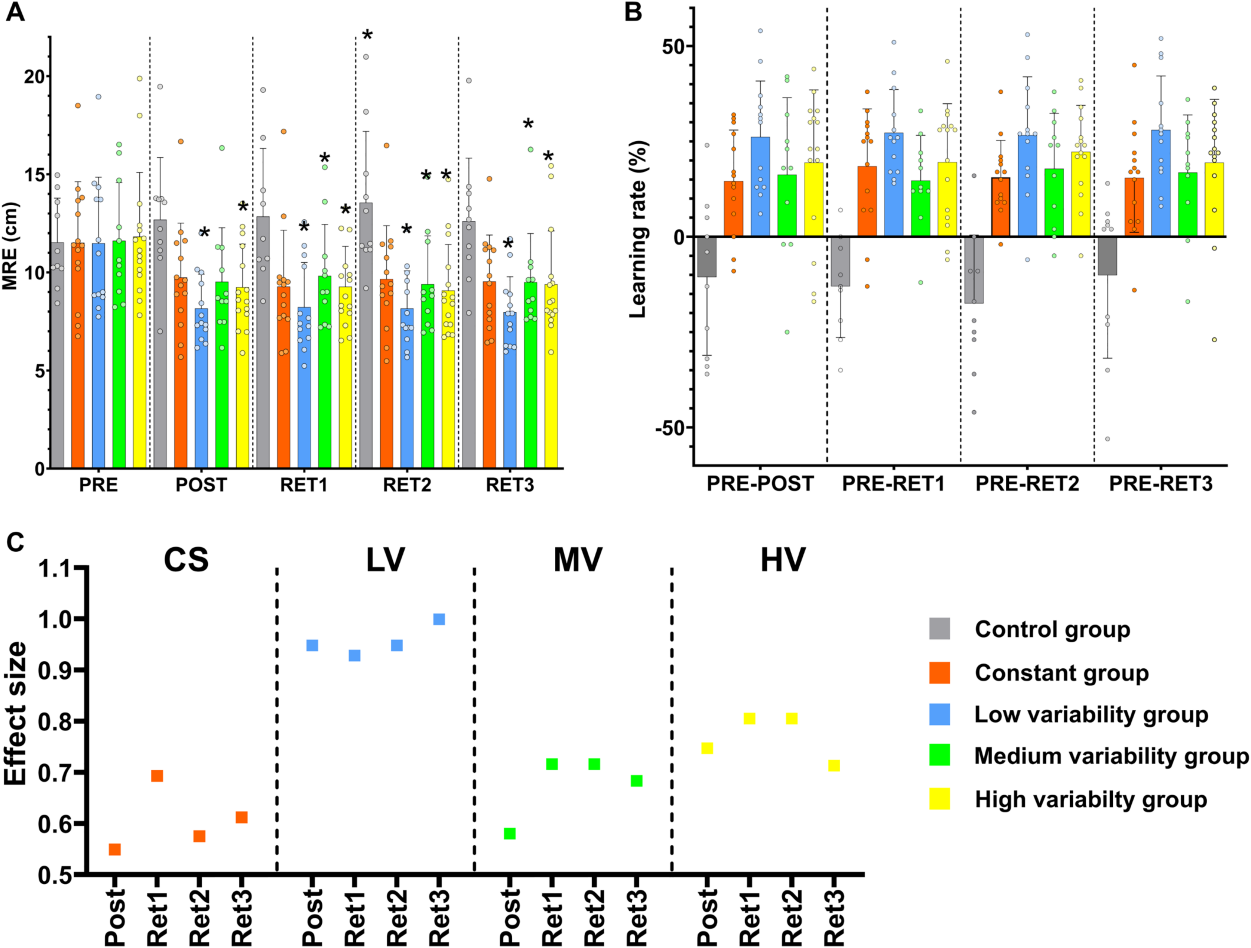
Mixed ANOVA, residual amount of learning and effect size results. (A) Pairwise comparisons between tests in all variables for all the groups. * = significant differences compared to the pre-test; (B) Plot displaying the residual amount of learning; (C) Effect sizes representation for pair comparisons between the evaluation tests in all variables for all the practice groups. CS = constant group; LV = low variability group; MV = medium variability group; HV = high variability group; MRE = mean radial error; Post = comparison between the pre-test and the post-test; Rt 1 = comparison between the pre-test and the retest 1; Rt 2 = comparison between the pre-test and the retest 2; Rt 3 = comparison between the pre-test and the retest 3.

Note that the database, including the error computed by axes separately, is available in the Supplemental Material. Means and standard deviations for groups in all tests are displayed in Table 2 and Fig. 4.

**Table 2:** Average error values of the MRE (mean ± SD) for every group in all the tests calculated in the study.

Variable	Groups	Pre-test	Post-test	Retest 1	Retest 2	Retest 3
MRE (cm)	C	11.53 ± 2.26	12.69 ± 3.17	12.85 ± 3.47	13.55 ± 3.63	12.61 ± 3.21
	CS	11.52 ± 3.11	9.74 ± 2.76	9.28 ± 2.87	9.66 ± 2.72	9.54 ± 2.36
	LV	11.48 ± 3.37	8.16 ± 1.75	8.23 ± 2.28	8.16 ± 1.92	7.98 ± 1.80
	MV	11.61 ± 2.97	9.53 ± 2.75	9.82 ± 2.62	9.40 ± 2.46	9.50 ± 2.48
	HV	11.82 ± 3.28	9.24 ± 2.20	9.27 ± 2.05	9.07 ± 2.34	9.39 ± 2.72

**Note:**

MRE = Mean radial error; C = control group; CS = constant group; LV = low variability group; MV = medium variability group; HV = high variability group.

The mixed ANOVA performed with all the groups showed a significant interaction effect (MRE, F_12.268,117.879_ = 4.630, *p* < 0.001). This significant interaction was led by a clear difference between the control group and the rest of the groups. The control group displayed significantly worse performance results in the post-test (12.7 ± 3.2 cm) compared to the pre-test (11.5 ± 2.3 cm) and this performance detriment was maintained in the rest of evaluations (Retest 1 = 12.8 ± 3.5 cm; Retest 2 = 13.5 ± 3.6 cm; Retest 3 = 12.6 ± 3.2 cm). However, the experimental groups improved their performance significantly when the initial values from the pre-test (CS = 11.5 ± 3.1 cm; LV = 11.5 ± 3.4 cm; MV = 11.6 ± 3 cm; HV = 11.8 ± 3 cm) were compared to the rest of evaluations (see Fig. 4). Specifically, all the practice groups aside the MV group showed significant differences between the pre-test and the post-test (Post: CS = 9.7 ± 2.8 cm; LV = 8.2 ± 1.7 cm; MV = 9.5 ± 2.7 cm; HV = 9.2 ± 2.2 cm); all the practice group displayed significant differences between the pre-test and the retest 1 (Retest 1: CS = 9.3 ± 2.9 cm; LV = 8.2 ± 2.3 cm; MV = 9.8 ± 2.6 cm; HV = 9.3 ± 2 cm); in addition, all the practice group displayed significant differences between the pre-test and the retest 2 (Retest 2: CS = 9.7 ± 2.7 cm; LV = 8.2 ± 1.9 cm; MV = 9.4 ± 2.5 cm; HV = 9.1 ± 2.3 cm); and the same results were found comparing the pre-test and the retest 3 (Retest 3: CS = 9.5 ± 2.4 cm; LV = 8 ± 1.8 cm; MV = 9.5 ± 2.5 cm; HV = 9.4 ± 2.7 cm) (see Fig. 4A). No significant differences were found between the evaluations after practice. The fact that the control group was the only group that worsened supports that the training period was effective (see Table 2 for more details).

In order to check if there were significant differences between the practice groups hidden by the huge differences with the control group, another mixed ANOVA was performed only with the practice groups (CS, LV, MV, and HV). There were no statistical differences between groups. Effect sizes were computed to assess the magnitude of change in performance by practice in each group (Fig. 4C).

The differences between the pre-test, the post-test, and the retests for MRE showed large effect sizes for the low variability group (Hedge’s *g*: Pre-Post = 0.948; Pre-Retest 1 = 0.928; Pre-Retest 2 = 0.948; Pre-Retest 3 = 0.999) and moderate effect sizes for the rest of the experimental groups (Hedge’s *g*: CS–Pre-Post = 0.549; Pre-Retest 1 = 0.693; Pre-Retest 2 = 0.575; Pre-Retest 3 = 0.612; MV–Pre-Post = 0.580; Pre-Retest 1 = 0.716; Pre-Retest 2 = 0.716; Pre-Retest 3 = 0.683; HV–Pre-Post = 0.747; Pre-Retest 1 = 0.805; Pre-Retest 2 = 0.805; Pre-Retest 3 = 0.713) (Fig. 4C).

A correlational analysis was conducted to address how the initial intrinsic variability of participants could modulate the learning of the experimental task. Additional to the performance variables, the residual amount of learning (difference in MRE between the pre-test and retest 3 computed by a linear regression analysis–RAL), and the autocorrelation coefficient of the positions of the ball impacts in the pre-test were calculated in the four experimental groups (Table 3). Correlational analyses showed that the initial intrinsic variability measured through the autocorrelation of the time series data of error in the pre-test did not correlate with the residual amount of learning for the whole cohort (r = 0.055, *p* = 0.671). However, once the sample was split according to the training group, a positive correlation was found for the low variability group (r = 0.604; *p* = 0.29) (Table 4).

**Table 3:** Average values (mean ± SD) of the initial global autocorrelation and residual amount of learning, between the pre-test and the retest 3 for each group.

	Global autocorrelation	Residual amount of learning (cm)
C	0.07 ± 0.12	−2.99 ± 2.60
CS	0.01 ± 0.07	0.07 ± 1.36
LV	0.05 ± 0.11	1.62 ± 1.60
MV	0.01 ± 0.11	0.17 ± 1.69
HV	0.09 ± 0.08	0.40 ± 1.92

**Note:**

C = control group; CS = constant group; LV = low variability group; MV = medium variability group; HV = high variability group; C = control group.

**Table 4:** Correlations between the initial intrinsic variability and the residual amount of learning for each group.

	CS	LV	MV	HV
Pearson correlation	0.184	0.604*	0.296	−0.061
Sig.	0.529	0.029	0.377	0.830

**Note:**

* *p* < 0.05.

CS = constant group; LV = low variability group; MV = medium variability group; HV = high variability group.

## Discussion

The theoretical usefulness of enhancing motor variability through manipulating the practice conditions to promote a higher amount of learning has been previously addressed (Asencio-Alonso, Gea García & Menayo Antúnez, 2021; Hernández-Davo et al., 2014; Williams & Hodges, 2005; Zetou et al., 2014). However, the benefits of variable practice have not always been observed in experimental studies (Breslin et al., 2012; Harris & Wolpert, 1998; Keetch et al., 2005; Shmuelof, Krakauer & Mazzoni, 2012). From the authors’ point of view, the lack of adaptation of the training load of variable practice to the inherent individual features could be one of the main reasons for the controversial results. Most of the studies in the existing literature have compared constant practice *versus* variable practice. Although some studies have analyzed the effect of different variability scheduling, suggesting gradual increases in contextual interference for more effective motor learning (Porter & Beckerman, 2016; Porter & Magill, 2010), no experiments have compared different levels of heterogeneity or numerosity of variability (Raviv, Lupyan & Green, 2022). The load of variable practice was manipulated in this work through the heterogeneity of the throwing movements to analyze their effect on the learning process of a discrete throwing task.

### Do different loads of variable practice induce different amount of learning?

All the experimental groups improved their throwing performance after the intervention compared to the control group. Even though all practice conditions improved throwing accuracy, it was expected that the amount of learning would not be similar between groups. Therefore, which were the best conditions concerning variable practice to improve the learning process? One of the main ideas that guided this study is that applying variable practice could ease the individual’s exploratory behaviors or enhance the individual’s sensitivity to their own motor error, promoting adaptative behaviors and a faster learning rate (Barbado et al., 2017; Manor et al., 2010; Wu et al., 2014; Zhou et al., 2013). Inducing variability would provide learners with more opportunities to search and explore their environment to create a range of effective and adaptable movement solutions, leading to a higher probability of generalizing outside the movements practiced (Tenenbaum & Griffiths, 2001). However, according to the results of the current study, it cannot be confirmed that variable practice was better than constant practice since the variability groups did not show significantly better results than the constant group. It has been emphasized that in dynamic environments, conditions are never identical. The time or the space needed to perform a task continuously changes, whether the skill is performed in a high or low predictable environment. Therefore, based on the principle of ‘repetition without repetition’ (Bernstein, 1967), every movement is different, even in closed skills. Adapting to the changing conditions provided by the neuromuscular system in the interaction with the environment is a key factor for success. In this sense, previous researchers have highlighted the need to adjust the amount of variability through the manipulation of individual, environmental, and task constraints. Renshaw et al. (2016) suggest the need to find the optimum level of variability. They proposed that a low amount of variable practice would not promote additional motor pattern forming or an adequate system reorganization, but, on the other hand, too much variability would make the environment unmanageable for the learner.

Based on the rationale mentioned above, it was hypothesized it would be possible to observe an inverted U-Shape between the different loads of variable practice and the amount of learning in adaptation (post-test) and consolidation tests (retests). Therefore, it was checked if low-to-medium levels of variability could be optimal to improve the amount of learning as suggested by previous studies (Caballero, Luis & Sabido, 2012; Harbourne & Stergiou, 2009; McEwen & Lasley, 2002; Moreno & Ordoño, 2015; Ranganathan & Newell, 2010). According to the findings of this study, no significant differences in the amount of learning were observed between practice groups, so initially, the inverted U-Shape cannot be confirmed. Nevertheless, from the authors’ point of view, the fact that the low variable practice group showed larger benefits in terms of effect size (0.93 ≤ d ≤ 1.00) compared to the other group (Constant group: 0.55 ≤ d ≤ 0.69; Medium variability group: 0.58 ≤ d ≤ 0.72; High variability group: 0.71 ≤ d ≤ 0.80) makes it not entirely possible to reject the hypothesis. Figure 4C shows that the lowest variability conditions (*i.e*., constant practice group) and the highest variability conditions (*i.e*., high variability and even medium variability groups) did not cause such a large learning effect size in this throwing task. The lower effect size observed in the constant practice group could be caused by a reduced practice stimulus that did not maximize the learning process. Therefore, some increased level of motor variability seems to be helpful in improving the learning process. Supported by the idea that the CNS regulates intrinsic motor variability to enhance the ability to adapt (Barbado et al., 2012; Davids et al., 2003; Lamoth & van Heuvelen, 2012; Mandelblat-Cerf, Paz & Vaadia, 2009; Moreno & Ordoño, 2010; Wu et al., 2014; Zhou et al., 2013), the variable practice could stimulate the intrinsic variability to keep exploring the possible solutions. However, the results from the medium and high variability groups do not support the application of high levels of variability to foster the learning process. Based on these results, high variable practice conditions might hinder the search for a stable motor solution to fulfill the demands of the throwing task proposed in this study. In spite of these noticeable findings, it must be reinforced that the sample size does not allow us to confirm the inverted U-shape hypothesis. Therefore, more research is needed to elucidate whether there is an optimal variability load for promoting a higher amount of learning depending on the task and individual features.

### Do the individual’s intrinsic features modulate the effect of variable practice?

Quite possibly, the differences found regarding the effect of the different levels of variability can be related to the fact that not all individuals respond in the same way to a similar training stimulus. Therefore, the critical point is that the individual’s characteristics modulate the role of motor variability during the learning process (Caballero et al., 2017; Newell & Vaillancourt, 2001; Vaillancourt & Newell, 2002, 2003). Renshaw et al. (2016) proposed that the amount of variable practice needed to be matched to the performer’s skill level. Beginners could benefit from low loads of variable practice, which guide their exploration towards reduced functional solutions. Conversely, the most expert performers would need more significant enhanced variability during practice to promote more dexterous behaviors (García-Herrero et al., 2016). Another important learner feature is the intrinsic motor variability itself. Previous studies have shown that a higher motor variability (*i.e*., higher motor exploration) at the baseline is related to a higher amount of learning (Barbado et al., 2017; Wu et al., 2014). Based on this idea, it could be expected that people with higher intrinsic motor variability do not need additional variable practice to promote a higher amount of learning. On the other hand, people with low intrinsic motor variability would benefit from variable practice.

In this study, the motor task performed was a relatively common throwing and catching ball task, but participants displayed different initial performance levels as well as different initial intrinsic variability. The participants were distributed into the experimental groups so there were no differences between groups at the beginning of the treatment. Nonetheless, it also implies that, in every group, there were participants with a different initial skill level and different initial intrinsic variability, so this should have been considered. The correlational analyses showed that those participants with higher errors in the pre-test showed a higher amount of learning. Thus, the amount of learning is highly determined by the initial performance because less skillful individuals have more room for improvement than the more skilled ones. With the purpose of avoiding the potential bias on the amount of learning caused by the initial performance, a residual amount of learning was computed using a linear regression method (Barbado et al., 2017; Caballero, Moreno & Barbado, 2021) (see the Statistical Analysis section for more details). The participants’ initial intrinsic variability by the global autocorrelation of the time series data of the ball position was also measured, providing information about the internal relationships between the successive attempts to hit the target. Bivariate correlational analyses showed that more autocorrelated variability was not related to the amount of learning when all the participants, independently of the training group, were included in the analysis. However, once the sample was separated according to the training groups, those variables were positively correlated for the group in which participants trained with a low load of variable practice. The autocorrelation of the error time series data has been used to identify the way in which the performer varies trying to adjust his/her movement to an external target, and it has been related to the intention to adjust the movement to a target (Urbán et al., 2019). In this sense, the behavior of individuals who showed higher autocorrelated fluctuations (*i.e*., lower number of changes in the error direction) performed fewer movement adjustments (Amoud et al., 2007; Wang & Yang, 2012; Zhou et al., 2013), which in turn, would be an index of poor explorative behavior or lower motor error sensitivity (Barbado et al., 2017). Based on this feature, these participants would be more benefited by variable practice, to promote intrinsic exploration behavior. A variable environment would help them find an optimal motor solution to adjust their movement to the target and foster their learning. These results are in coherence with those found in an advanced stage of learning, in which exploitation rather than exploration behavior is utilized (Herzfeld & Shadmehr, 2014; Wu et al., 2014). It must be pointed out that this correlation was not observed when higher variable practice was applied (MV and HV groups). In these cases, individuals with lower intrinsic exploration behavior did not benefit by an “excessive” variable practice. These results also seem to support the idea that an optimal load of variable practice must be found to maximize the amount of learning according to each participant’s features. However, this rationale should be confirmed by future studies as the low sample size for each group could bias the correlation results.

### Limitations

The conclusions of this study have to be considered with caution due to some limitations. The main limitation was the small sample size, making it difficult to find differences between groups despite the large effect sizes observed. Additionally, the groups were balanced according to the initial performance of the participants, but groups were heterogeneous in the distribution of the autocorrelated variability of the participants. Considering this and following previous suggestions (Woolley & Doupe, 2008; Wu et al., 2014), a larger sample size would have allowed the groups to be divided according to their intrinsic variability and performance levels to confirm how the intrinsic characteristic of the learner could mediate the effect of the different load of variable practice.

In order to diminish the attentional demands that can be attributed to selecting the targets to throw at in the variable practice conditions, the targets were carefully numbered and colored to facilitate their identification. Nevertheless, it can be considered an additional limitation of the study.

Another limitation is related to the fact that we did not assess the performance during the learning practice period, as well as the intrinsic variability within each experimental group throughout the acquisition phase. The evolution of performance during practice can be informative about the participants’ learning processes within each group. However, it was not assessed as the performance during practice trials could not be compared because each group practiced throwing toward different dispersed targets. The intrinsic variability of the participants could be used to analyze the extent of variability load they were exposed to, and it could be considered in future studies.

It also has to be taken into consideration that in this experiment, the load of variable practice has been chosen arbitrarily, according to the limits and possibilities of the task. The highest level was determined by the maximal angle in throwing that allowed the participants to catch the ball repeatedly without moving their feet from the marks on the floor. Therefore, the classification of low, medium, and high variability levels has to be taken with caution because these were not adjusted to the level of the learner. Moreno & Ordoño (2015) proposed that the tasks should be designed with a load level higher than the demands to which the learner is adapted to facilitate a new level of adaptation. To the best of the authors’ knowledge, no previous literature has addressed objective procedures to measure the load of variable practice in experimental learning procedures. Future studies should be directed to manipulate the variable practice level adapted to the learner’s intrinsic variability.

Finally, a possible relation between the effect of different loads of variable practice and the initial intrinsic variability of the learner measured by the autocorrelation of the time series data from the error of their trials has been found. The autocorrelation function measures linear dependencies between data points but not nonlinear dependencies. It cannot distinguish the structure of the variability to allocate the complex, chaotic, or random characteristics (Hunt, 2016). Due to the time series data length analyzed, other nonlinear tools that assess long range autocorrelation, like detrended fluctuation analysis, were not used. Nevertheless, linear autocorrelation can be used to analyze the fluctuations and dependencies of data points in a time series and it is applicable in short time series data.

## Conclusions

The results of the current study do not support the idea that variable practice is better than constant practice because medium and high variability groups did not show significantly better results than the constant group. Nevertheless, different loads of variable practice seem to induce different performance improvements in a throwing task. Although no significant differences in performance were observed at the last retest between the experimental groups, the degree of improvement quantified by the effect sizes showed that the group in which the low level of variability was applied showed higher amount of learning.

This finding supports the idea that variable practice seems to be a key factor to optimize motor learning if the variable practice load is manipulated according to the participants’ intrinsic motor variability. Specifically, learners with poor explorative behavior would benefit from the application of moderate loads of variable practice.

## Additional Information and Declarations

### Data Availability

The following information was supplied regarding data availability: The data is available at figshare: Caballero, Carla; Barbado, David; J. Moreno, Francisco (2022). Database of Practice design with different levels of variability. figshare. Dataset. https://doi.org/10.6084/m9.figshare.20039429.v2.
